# 567. Reasons for Deferral of COVID-19 Vaccines Among Arab American Healthcare Professionals Living in the United States

**DOI:** 10.1093/ofid/ofab466.765

**Published:** 2021-12-04

**Authors:** Anita Shallal, Evi Abada, Rami Musallam, Omar Fehmi, Linda Kaljee, Ziad Fehmi, Suma Alzouhayli, Deema Ujayli, Doreen Dankerlui, Seongho Kim, Vijaya Arun Kumar, Marcus Zervos, Rouba Ali

**Affiliations:** 1 Henry Ford Hospital, Detroit, MI; 2 Detroit Medical Center / Wayne State University, Detroit, MI; 3 Wayne State University, Detroit, MI; 4 n/a, Detroit, MI; 5 Henry Ford Health System, Detroit, MI; 6 Detroit Medical Center/Wayne State University, Detroit, MI; 7 Michigan State University, Detroit, MI; 8 Karmanos Cancer Institute, Detroit, MI; 9 Wayne State University School of Medicine, Detroit, MI

## Abstract

**Background:**

The WHO identified the three most common reasons for worldwide vaccine hesitancy to be safety concerns, lack of knowledge and awareness, and religion and cultural issues. There is limited information on this topic among Arab Americans, a rapidly growing demographic in the US. We sought to determine the reasons for deferral of the coronavirus disease 2019 (COVID-19) vaccine amongst Arab American health professionals living in the US.

**Methods:**

This was a cross-sectional study utilizing an anonymous online survey. The survey was distributed via e-mail to National Arab American Medical Association members and Arab-American Center for Economic and Social Services healthcare employees. Respondents were considered vaccine hesitant if they selected responses other than a willingness to receive the COVID-19 vaccine.

**Results:**

A total of 4,000 surveys were sent via e-mail from December 28 2020 to January 31 2021. The highest group of respondents were between the ages of 18-29 years and physicians constituted 48% of the respondents. Among 515 respondents, 41.9% (n=216) would receive the vaccine within one month of it becoming available to them, and 30.2% (n=156) had already received a vaccine. Among those who would defer the vaccine, 9.3% (n=48) would receive it within 1-3 months, 5.6% (n=29) within 3-6 months and 6.6% (n=34) after over 6 months or longer. 6.2% (n=32) would not receive the vaccine. The three most commonly reported reasons for deferral of vaccine among 75 vaccine hesitant respondents were: “I am worried about the side effects” (65.3%), “I am worried the vaccine moved through clinical trials too fast (54.7%), and “There is no information about long term side effects of the vaccine” (52%). Data indicate that about a quarter of respondents also expressed distrust of the government and the pharmaceutical industry. The results are summarized in table 1.

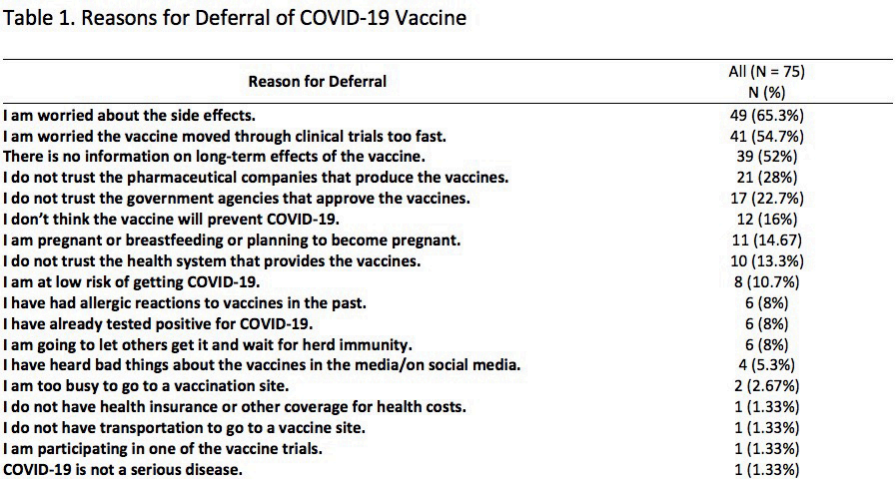

**Conclusion:**

Reasons cited by this sample of Arab Americans for deferring the COVID-19 vaccine mirror more general concerns about vaccine side effects and need for information. Concerns about clinical trial procedures and distrust have become more prevalent with COVID-19. This data can help inform COVID-19 vaccine advocacy efforts among health care providers, and thus could have substantial impact on vaccine attitudes of the general population.

**Disclosures:**

**Marcus Zervos, MD**, **contrafect** (Advisor or Review Panel member)**janssen** (Grant/Research Support)**merck** (Grant/Research Support)**moderna** (Grant/Research Support)**pfizer** (Grant/Research Support)**serono** (Grant/Research Support)

